# Acoustic differentiation and classification of wild belugas and narwhals using echolocation clicks

**DOI:** 10.1038/s41598-021-01441-w

**Published:** 2021-11-12

**Authors:** Marie J. Zahn, Shannon Rankin, Jennifer L. K. McCullough, Jens C. Koblitz, Frederick Archer, Marianne H. Rasmussen, Kristin L. Laidre

**Affiliations:** 1 School of Aquatic and Fishery Sciences, University of Washington, 1122 NE Boat Street, Seattle, WA 98105 USA; 2 Southwest Fisheries Science Center, NOAA, 8901 La Jolla Shores Drive, La Jolla, CA 92037 USA; 3 Pacific Islands Fisheries Science Center, NOAA, 1845 Wasp Boulevard, Building 176, Honolulu, HI 96818 USA; 4 Max Planck Institute of Animal Behavior, Advanced Research Technology Unit, Konstanz, Germany; 5 Centre for the Advanced Study of Collective Behaviour, University of Konstanz, Konstanz, Germany; 6 Department of Biology, University of Konstanz, Konstanz, Germany; 7 The University of Iceland’s Research Center in Húsavík, Húsavík, Iceland; 8 Polar Science Center, Applied Physics Laboratory, University of Washington, 1013 NE 40th Street, Seattle, WA 98105 USA

**Keywords:** Marine mammals, Conservation biology, Ecological modelling

## Abstract

Belugas (*Delphinapterus leucas*) and narwhals (*Monodon monoceros*) are highly social Arctic toothed whales with large vocal repertoires and similar acoustic profiles. Passive Acoustic Monitoring (PAM) that uses multiple hydrophones over large spatiotemporal scales has been a primary method to study their populations, particularly in response to rapid climate change and increasing underwater noise. This study marks the first acoustic comparison between wild belugas and narwhals from the same location and reveals that they can be acoustically differentiated and classified solely by echolocation clicks. Acoustic recordings were made in the pack ice of Baffin Bay, West Greenland, during 2013. Multivariate analyses and Random Forests classification models were applied to eighty-one single-species acoustic events comprised of numerous echolocation clicks. Results demonstrate a significant difference between species’ acoustic parameters where beluga echolocation was distinguished by higher frequency content, evidenced by higher peak frequencies, center frequencies, and frequency minimums and maximums. Spectral peaks, troughs, and center frequencies for beluga clicks were generally > 60 kHz and narwhal clicks < 60 kHz with overlap between 40–60 kHz. Classification model predictive performance was strong with an overall correct classification rate of 97.5% for the best model. The most important predictors for species assignment were defined by peaks and notches in frequency spectra. Our results provide strong support for the use of echolocation in PAM efforts to differentiate belugas and narwhals acoustically.

## Introduction

**Figure 1:**
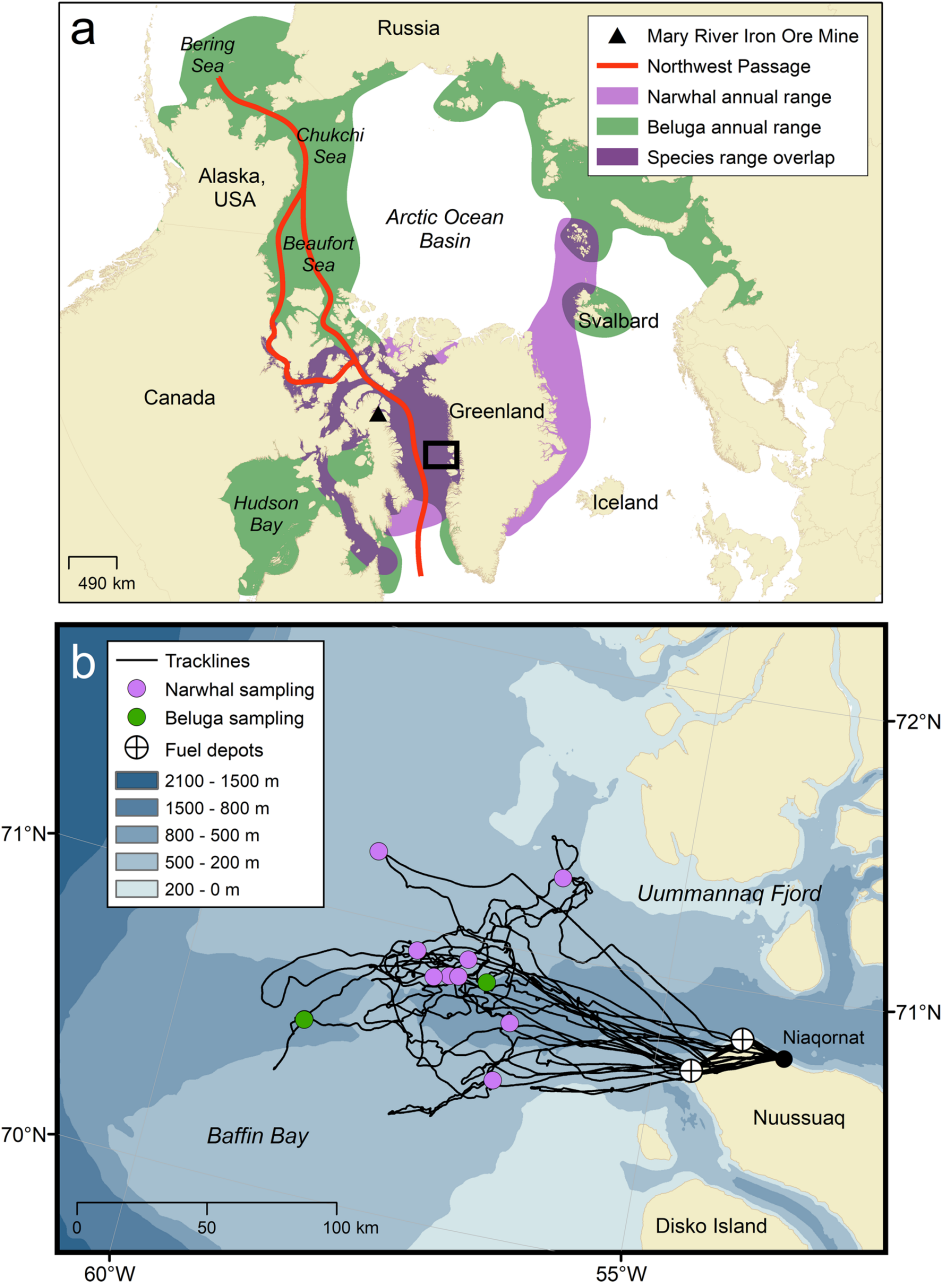
Top panel: map of narwhal (purple) and beluga (green) annual ranges (**a**). Overlapping beluga and narwhal annual ranges is visible in dark purple. The Northwest Passage sea route between the Atlantic and Pacific oceans is shown in red. Bottom panel: map showing track lines of search effort, fuel depots, and recording sites for narwhals and belugas in Baffin Bay, West Greenland, between March 21st and 31st, 2013 (**b**). The black inset box in (**a**) demarcates the area shown in (**b**). Maps were created using ArcGIS’s ArcMap software (v. 10.8; https://www.esri.com/en-us/arcgis/products/arcgis-desktop/resources).

Only three species of cetaceans occupy the Arctic year-round: the beluga (*Delphinapterus leucas*), narwhal (*Monodon monoceros*), and bowhead whale (*Balaena mysticetus*). As toothed whales (odontocetes), the beluga and narwhal are closely related and are the only two members of the Monodontidae family. They use echolocation to identify objects and locate prey, unlike the bowhead whale, a baleen whale, that has not evolved this sense^1,2^. Belugas are circumpolar in their distribution with approximately 22 subpopulations, or stocks, some of which are highly migratory and others resident in both Arctic and sub-Arctic waters^3–9^. Most populations migrate from wintering regions among the pack ice and return to the same estuarine summering areas to feed, molt, and give birth^8,10,11^. In contrast, narwhals occur in approximately 12 stocks and have a more restricted distribution occupying waters of the Canadian Arctic, West and East Greenland, and western Russia within the Atlantic Arctic^12–14^. Narwhals in the Canadian Arctic and West Greenland undergo extensive annual migrations with high site fidelity from their summer ranges in fjords of Greenland and Baffin Island to their wintering grounds in Baffin Bay and northern Davis Strait^3,13,15^. Across their respective distributions, belugas and most of the world’s narwhals overlap for much of the year in the waters of the Canadian Arctic and Baffin Bay, West Greenland, during their annual migrations (Fig. 1a).

In addition to the use of visual surveys and telemetry to study wild beluga and narwhal populations, Passive Acoustic Monitoring (PAM) uses hydrophones over large spatiotemporal scales to localize regions of ecological importance^12,16–20^. Despite this, the acoustic profiles of Arctic odontocetes remain largely understudied, particularly during non-summer months, because they reside under sea ice much of the year and the deployment and recovery of acoustic equipment in the Arctic is challenging and costly^12^. Further, PAM relies on the ability to distinguish between species solely based on their characteristic sounds (i.e., calls or echolocation clicks), which means individual or a combination of specific sonic identifiers must be known for each species. Call types typically used in acoustic classification of odontocetes include echolocation clicks, burst pulses, whistles, and combined signals^20–26^. Echolocation, sometimes referred to as biosonar, is characterized by the emission of high frequency, relatively broadband clicks of high directionality and listening for returning echoes; it is a dominant sense for odontocetes, much like vision is for humans^1,2^. Burst pulses, or “buzzes,” are short bursts or a series of broadband pulses (clicks) with a high repetition rate^21,25,27^. Whistles are narrow-band and frequency-modulated tonal vocalizations^21,28^. Finally, combined signals, or “mixed calls,” include overlaid or paired pulsed and tonal sounds^23,29,30^. Acoustic classifiers may use a single call type or multiple call types to differentiate species^31^.

Belugas and narwhals have large vocal repertoires that feature similar acoustic profiles^17,23,27,32^, which can make it difficult to distinguish between them acoustically without visual confirmation of species identity. To date, only one study has differentiated between belugas and narwhals acoustically^17^ since other PAM studies have focused on regions where they do not co-occur^12,33,34^. Frouin-Mouy et al*.*^17^ identified narwhals when they observed whistles or buzzes combined with low-frequency echolocation clicks, and belugas were detected when bird-like whistles were combined with high-frequency clicks. When whistles and buzzes were absent, they used an increase in spectral power around the 20 kHz frequency band to identify narwhal mid-frequency clicks^17^. However, their comparison was narrow in scope as it was not their primary research objective. Thorough analyses of echolocation clicks have been conducted separately on belugas^21,36–40^ and narwhals^25,27,41,42^, but no comparative study with data from the same region exists. The broadband signals of beluga and narwhal echolocation extend frequencies between 2–150 kHz, but high-frequency clicks have been reported for both species with energies up to 200 kHz^25,39^. Measurements of beluga click peak frequencies in captive and wild environments vary widely between 40–120 kHz^16,36–39^. Unlike belugas, narwhals have not been held in captivity where controlled experiments can occur, and as a result, narwhal phonation is poorly described compared to belugas and other delphinids. Narwhal echolocation has been characterized by clicks with frequency maxima between 30–70 kHz^25,30,43,44^, with some as low as 2–10 kHz and 7–14 kHz^32^. Still, among these studies, differences in study design, sampling equipment, and location make direct comparisons between beluga and narwhal click characteristics challenging.

As the Arctic continues to experience profound environmental changes due to climate warming^45–47^, monitoring changes to the seasonal presence of belugas and narwhals is a growing research priority. Projections suggest that trans-Arctic shipping routes like the Northwest Passage and Northern Sea Route will be ice-free by midcentury^48,49^, leading to concerns surrounding the effects of increased human activities on Arctic cetaceans^50,51^. Importantly, the spatiotemporal overlap of belugas and narwhals also overlaps with the vessel corridor for the Mary River Iron Ore Mine on Baffin Island and the Northwest Passage (Fig. 1a) where potentially large increases in underwater noise are expected in the coming decades^3,52–54^. Arctic odontocetes are especially at risk to northward human expansion as hydrocarbon development and commercial shipping pose substantial threats via ship strikes, hearing damage, vessel disturbance, and increased underwater noise^3,50,51^. Underwater anthropogenic sounds from seismic surveys, drilling and oil production, military sonar, and motorized vessels can substantially disturb the acoustic environment which they depend on^53,55–59^.

Here, we use data collected from an offshore region in Baffin Bay, West Greenland to compare beluga and narwhal echolocation clicks. Our research objectives were twofold: (1) determine whether spectral properties between beluga and narwhal echolocation are significantly different, and (2) build an acoustic classifier to determine whether belugas and narwhals can be classified using only echolocation parameters. Employing methods to differentiate belugas and narwhals acoustically will equip scientists with the necessary tools to monitor their distribution and habitat-use changes. Further, using PAM to study Arctic odontocetes year-round will help managers mitigate any negative consequences of climate change and vessel traffic on these sentinel species.

## Methods

### Data collection

Aerial searches for narwhals and belugas were conducted out of Niaqornat, West Greenland, from an Air Greenland AS350 helicopter in spring 2013 (Fig. 1b). All field operations were in accordance with IACUC procedures as approved by the University of Washington (#4155-01, PI Laidre) and the US Office of Naval Research. The Government of Greenland and Greenland Institute of Natural Resources, Nuuk provided K. L. Laidre permission to conduct research in Greenland waters. All methods were carried out in accordance with relevant guidelines and regulations. Weather conditions allowed seven days between 21 and 31 March to search for whales 100–150 km offshore in leads and cracks in the pack ice > 98% concentration. When narwhals or belugas were observed, an aerial search radius of at least 5 km was required to ensure that the ice conditions were safe for landing, during which sightings for other whales and species in the vicinity were made. On the ice, a hydrophone array was deployed at the edge of a lead and all recordings were paired with visual identification of each species. Only one species was observed and recorded at each sampling location, so there were no occurrences in which both narwhals and belugas were present. Since the detection range for echolocation is < 1 km^39^ and our search radius was > 5 km, we assume our data are single-species recordings.

The hydrophone array was composed of 16 individual Reson TC4013-5 receivers (sensitivity − 215 dB ± 2 dB re 1 V/µPa; flat ± 2 dB frequency response 1–150 kHz) positioned in a vertical, linear orientation. Prior to deployment, each hydrophone was calibrated and its frequency response determined. Each receiver was located 1 m apart along a 2 mm diameter line with the topmost hydrophone at 3 m below the surface and the bottom hydrophone at 18 m. A 4-kg weight attached to the bottom maintained a vertical orientation of the array. While recording, a custom software, MALTA (Microphone Array Localization Tool for Animals by CAE Software and Systems), was used to visually examine recordings of all 16 receivers in real-time. Hydrophone signals were amplified by 35 dB using a custom amplifier, and recordings were converted from analog to digital (500 kHz sampling rate; 16-bit resolution) using two eight-channel National Instruments PXI-6123 A/D converters. No high pass filter was applied, and the hydrophones served as a low pass filter (150 kHz, 1 pole). The clipping level was at 206 dB pp re 1 µPa at 100 kHz^39,41^. To safeguard against potential file corruption and facilitate data post-processing, whale recordings were partitioned, loss-less, in 5-s-long sound files.

### Event selection and acoustic parameter estimation

Individual echolocation clicks were detected using the open source passive acoustic analysis software PAMGuard (v. 2.01.03f)^60^. Based on localization analyses using the same data^39,41^, the highest amplitude signals were recorded on hydrophones 10 and 11 (positioned at 12 and 13 m deep, respectively) and thus provided the highest quality recordings. For species differentiation analyses and classification models, only data from clicks recorded on hydrophone 10 (12 m depth) were used to avoid pseudo-replication. Using the Click Detector module in PAMGuard, clicks were detected from hydrophone 10 recordings with a 14 dB signal-to-noise minimum threshold. To minimize false detections on low-frequency sounds, the detector included a 4th order IIR Butterworth high-pass filter with a 4 kHz corner frequency. No additional frequency filters were used. Click detections were labeled using PAMGuard click classifiers that were defined by specified frequency bins according to the peak frequency within each click. Each classifier was given a unique numeric code that corresponded to the target detection frequency range: 1 (4–20 kHz), 2 (20–50 kHz), 3 (50–70 kHz), 4 (70–100 kHz), 5 (100–150 kHz), 6 (150–250 kHz), and 0 (unclassified).

Groups of clicks (i.e., click trains) were then manually assigned to individual detection “events” using the bearing/time display in PAMGuard’s Viewer Mode (v. 2.01.03f). Although data from only one hydrophone were necessary for our analyses, bearing angles produced from two channels (hydrophones 10 and 11) were needed to effectively visualize and isolate click trains. Since the classification models employed in downstream analyses utilized subsamples of the entire dataset to evaluate model performance—much like a testing and training dataset—we assigned clicks to acoustic events using predetermined time windows to facilitate random partitioning of the data across all recordings. Two-minute intervals were used for narwhal recordings and 1-min intervals were used for beluga recordings in an effort to obtain a similar number of clicks per acoustic event between species, because the beluga recordings had more overlapping click trains. For all events, we included clicks where the maximal intensity of the beam was both centered (i.e., on-axis) and not centered (i.e., off-axis) on the recording system to reflect the nature of most PAM data. Once all clicks were assigned to events, a suite of acoustic parameters was calculated (Table 1). Using the *PAMpal* package (v. 0.12.6)^61^ in R (v. 4.1.0)^62^, echolocation parameter values were calculated for the click sequences selected in PAMGuard using standard measurement criteria^63–65^. Default *PAMpal* settings were used that included a 10 kHz Butterworth high-pass filter and a FFT window length of 2.5 ms to use in calculations. Parameter values were calculated for individual clicks and then the mean parameter values determined for a given event, except for inter-click-interval (ICI) where the mode value was approximated for each acoustic event.

**Table 1:** Descriptions of echolocation acoustic parameters used as variables in species differentiation analyses and classification models. All parameters outlined here were used for the standard Random Forests classification model and BANTER’s first stage call classifier. Variable codes marked with an asterisk (*) indicate which acoustic parameters were used for multivariate differentiation analyses. A full list and the process used by the *PAMpal* package^61^ for calculating click parameters can be found in the R package documentation^65^.

Variable code	Variable name	Unit	Description
peak*	Peak frequency	kHz	Frequency value with the highest dB level
peak2*	Second peak frequency	kHz	Frequency value with the second highest dB level
peak3*	Third peak frequency	kHz	Frequency value with the third highest dB level
trough*	Frequency trough	kHz	Frequency value between peak and peak2 with the lowest dB value; also called spectral notch
trough2*	Second frequency trough	kHz	Frequency value between peak2 and peak3 with the lowest dB value
peakToPeak2*	First peak to second peak	kHz	Difference between the frequency values of peak and peak2
peakToPeak3*	First peak to third peak	kHz	Difference between the frequency values of peak and peak3
peak2ToPeak3*	Second peak to third peak	kHz	Difference between the frequency values of peak2 and peak3
Q_10dB	− 10 dB resonant quality factor	kHz	Metric of frequency pureness at − 10 dB calculated by dividing the center frequency by the bandwidth
Q_3dB*	− 3 dB resonant quality factor	kHz	Metric of frequency pureness at − 3 dB calculated by dividing the center frequency by the bandwidth
fmin_10dB	− 10 dB frequency minimum	kHz	− 10 dB frequency minimum
fmin_3dB*	− 3 dB frequency minimum	kHz	− 3 dB frequency minimum
fmax_10dB	− 10 dB frequency maximum	kHz	− 10 dB frequency maximum
fmax_3dB*	− 3 dB frequency maximum	kHz	− 3 dB frequency maximum
BW_10dB	− 10 dB bandwidth	kHz	− 10 dB bandwidth
BW_3dB*	− 3 dB bandwidth	kHz	− 3 dB bandwidth
centerHz_10dB	− 10 dB center frequency	kHz	[min frequency + (max frequency − min frequency)]/2 at − 10 dB
centerHz_3dB*	− 3 dB center frequency	kHz	[min frequency + (max frequency − min frequency)]/2 at − 3 dB
duration*	Click duration	µs	Time defined by the number of samples above 100 times the 40th percentile of the Teager Kaiser energy level
ici*	Inter-click interval	s	Time interval between consecutive clicks; mode approximated for each event

### Species differentiation

Multivariate analyses were used to assess potential differences in beluga and narwhal echolocation. When both the − 3 dB and − 10 dB measurement was calculated for acoustic parameters, only − 3 dB parameters were considered for differentiation analyses to remove redundant variables (see Table 1). To ensure − 10 dB measurements did not have a substantial contribution, we ran our analyses with both − 3 dB and − 10 dB measurements and our results were unaffected. Each acoustic parameter across all acoustic events was *z*-score transformed and a Euclidean distance dissimilarity matrix generated. A permutational multivariate analysis of variance (PerMANOVA; 999 permutations) was performed to test for differences between species among mean acoustic parameter values^66^. However, a significant result from a perMANOVA can result from differences in the mean positions of each group in multivariate space, differences in within-group variance in multivariate space, or a combination of the two. Therefore, a permutation test of multivariate homogeneity of dispersions (PERMDISP; 999 permutations) was used to analyze whether within-group variance in multivariate space between beluga and narwhal acoustic parameter values was different^67,68^. Differences between beluga and narwhal acoustic parameter values were visualized using principal component analysis (PCA) performed on a correlation matrix^69^. As an ordination technique, PCA reduces a high-dimensional dataset with correlated variables into fewer, uncorrelated dimensions called principal components (PCs)^70^. PCA variable loadings, or eigenvectors, on each PC were used to explore differences among groups. PCA eigenvalues provide the amount of variance explained by each PC and are used to determine which PCs are statistically significant. By applying the broken-stick rule and examining a scree plot, eigenvalues that are higher than what is expected by chance are considered to explain a significant amount of the variance in the original data^71^. All statistical analyses were conducted in R using the *vegan* package (v. 2.5-7)^72^.

### Species classification using Random Forests

We used a Random Forests (RF) classification model^73^ to classify belugas and narwhals using echolocation click parameters. RF has demonstrated to be an effective approach for bioacoustic studies^74–76^ as it is unaffected by nonparametric data and can accommodate many correlated variables. An RF model consists of many individual decision trees; each of these trees uses a random subset of samples and predictors. By combining thousands of trees, the final aggregated ensemble tree explores differences among species across the entire space of predictors in the original data and maximizes predictive power. RF models do not use a separate testing dataset to assess model accuracy. The random subsample drawn for each individual tree is termed the “in-bag” sample, while the remainder of the data not included are referred to as “out-of-bag” (OOB). OOB samples are used to test model performance by calculating an OOB classification error rate.

The two primary parameters for RF classification are the number of randomly selected predictors to choose from at each node, *mtry*, and the number of randomly selected samples to classify in each tree, *sampsize*. We conducted a sensitivity analysis for *mtry* and *sampsize* to ensure we used parameter values that prevented overfitting and maximized classification accuracy. The model was fit over all possible combinations of both parameters within possible ranges (*mtry*: 2–19; *sampsize*: 2–18) and the model accuracy determined. For all combinations, the correct classification rate did not vary by more than 0.05%, and therefore the model result was not sensitive to *mtry* and *sampsize*. Thus, we used the default value for *mtry* (square root of the number of predictors) and used half of the total sample size for the smallest species class for *sampsize*.

For our final model, species were assigned a priori to acoustic events in PAMGuard and 20 acoustic parameters were used as predictors to classify belugas and narwhals (Table 1). Each predictor (i.e., acoustic parameter) was the calculated mean across all individual clicks for each acoustic event. A randomized subset of four acoustic parameters (*mtry*) was used to split observations at each node. Each tree was grown using a randomized subset of nine acoustic events (*sampsize*) from each species group. The model was structured such that equal subsamples were drawn from each species class without replacement to account for our unbalanced dataset and to capture as much variation in the acoustic data as possible. For passive acoustics, there are occasions when individual whales may dominate the recordings with stereotyped calls; therefore, if we sampled with replacement, our model may underestimate the variation in the data. Model stability was visualized by plotting the trace of cumulative OOB error rate by number of trees and plotting the distribution of the fraction of trees that objects were in-bag. Ten thousand trees were constructed in the forest (*ntree*) to achieve model stability. To determine whether our model’s correct classification rates were significantly greater than what was expected by chance alone (50%), a binomial test for statistical significance of model performance was conducted. Our classification model was built using the *randomForest* (v. 4.6-14)^77^ and *rfPermute* (v. 2.5)^78^ packages in R.

### Species classification using BANTER

For comparison with the singular RF model above, we also employed BANTER (Bio-Acoustic eveNT classifiER), a supervised acoustic classification method that can utilize multiple call types^28^, to classify belugas and narwhals. BANTER consists of two stages of RF models^28^. The first stage is referred to as the “call classifier” in which individual classification models are built for each call type or detector. From the call classifier, BANTER produces a distribution of classification probabilities for each call type or detector. The second stage is the “event classifier” and uses the results from the first stage to classify acoustic events (collections of calls) to each species.

Although BANTER has the potential to use information from multiple call types (e.g., whistles or burst pulses), our objective was to determine whether belugas and narwhals can be classified using echolocation signals alone. Therefore, our call classifier consisted of separate RF models for each of the echolocation click detectors used in PAMGuard. Two click detectors (1 and 6) from the PAMGuard classification were removed due to insufficient data. For each detector, an RF model was built using 20 acoustic parameters (Table 1) to classify all individual echolocation clicks from that detector. RF models using default settings draw bootstrap samples from one pool containing the entire training dataset from all classes. Therefore, when an RF model is applied to an unbalanced dataset, the classifier will tend to correctly classify the dominant class. To offset this effect, BANTER draws equal random subsamples without replacement from each species group. Each model randomly selected 50 clicks from each species (*sampsize*) without replacement and each forest contained 20,000 trees. The number of parameters randomly selected to choose from at each node was set to the default (*mtry* = square root of the total number of parameters). Results from the call classifier provide mean assignment probabilities for each click detector. For example, the click detector 2 model produces the mean probability that clicks from detector 2 will be assigned to a beluga or narwhal. The call classifier also determines the proportion of each detector present for a given event. For example, all events that do not have clicks detected by click detector 2 will have a proportion of zero, and for events that do have clicks from detector 2 will have a proportion value between 0 and 1.

Results from the call classifier (i.e., mean assignment probabilities for each click detector and proportions of each detector per event) were then applied to the second stage event classifier as variables. The mode inter-click interval for each event was added as an additional event-level variable. The event classifier assigned all acoustic events to either a beluga or narwhal using a single RF model. Parameterization of this model was the same as the call classifier (*mtry* = default; *ntree* = 20,000), except the number of events randomly selected (*sampsize*) was set to nine. Relative variable importance was examined for both the call and event classifiers. To determine whether the correct classification rates of our BANTER call and event classifiers were significantly greater than what was expected by chance alone (50%), binomial tests for statistical significance were conducted for each model. Our BANTER classification model was built using the *banter* (v. 0.9.4)^28^ and *rfPermute*^78^ packages in R.

For the previous analyses, recordings were subdivided into 1- and 2-min acoustic events for belugas and narwhals, respectively. As an alternate approach to using standardized temporal intervals, we examined BANTER performance using events assigned to independent acoustic encounters. Acoustic encounters are defined by separate sightings of groups of whales and can vary in recording duration, ambient noise, and environmental and recording conditions (e.g., greater distance or different orientation between whales and receivers). Here, individual encounters were comprised of several acoustic events. If sounds from independent sightings were substantially different, encounters that have a larger number of events will have a greater representation in the species classification model used above. Using the same BANTER framework, we tested whether unique encounters for each species were similar. To accomplish this, a series of BANTER classification training models were created using all events from one encounter for each species (*mtry* = default; *ntree* = 10,000; *sampsize* = half the smallest class sample size) and then used to predict events from encounters that were not included. All unique pairwise combinations of acoustic encounters between species were used to build separate BANTER models. The correlation between the training confusion matrices and the prediction (validation) confusion matrices were then calculated. The presence of correlation (> 0.5) indicates that events from separate encounters are similar because the prediction classification scores are similar to the training scores.

## Results

A total of 1:03 h of beluga recordings and 7:38 h of narwhal recordings were made 100 km or more offshore in the pack ice of Baffin Bay (Fig. 1b). Belugas were observed and recorded at two unique locations and narwhals at nine unique locations, in pods of approximately six to thirty individuals. Narwhal recordings used for analyses originated from five independent sightings (i.e., encounters) and beluga recordings from two. We estimate there were approximately 22–36 individual belugas and 63–120 narwhals sampled across all seven encounters. Recordings were paired with visual confirmation of species, so all echolocation clicks were labeled as originating from belugas or narwhals. Species groups were assigned to events created in PAMGuard. A total of 11,319 clicks were assigned to 81 separate events with a median of 71 clicks per event (Supplementary Table S1). Out of the 81 events, 19 were belugas (2537 clicks) and 62 were narwhals (8782 clicks). Twenty acoustic parameters were used to compare beluga and narwhal echolocation (Table 1). Signal parameter measurements for each species across all acoustic events are summarized in Table 2. Beluga acoustic events had a higher mean peak frequency (68.7 ± 10.1 kHz), − 3 dB center frequency (69.6 ± 10.6 kHz), and − 10 dB center frequency (70.2 ± 9.2 kHz) than narwhal events (43.7 ± 7.8 kHz, 43.8 ± 7.8 kHz, and 46.2 ± 8.5 kHz, respectively). Spectral peaks, troughs, and center frequencies (variables: peak, peak2, peak3, trough, trough2, centerHz_10dB, and centerHz_3dB) for beluga clicks were generally > 60 kHz and narwhal clicks < 60 kHz with overlap between 40–60 kHz (Table 2).

**Table 2:** Summary of signal parameter measurements for beluga and narwhal echolocation for all 81 acoustic events (beluga: *n* = 19; narwhal: *n* = 62). Mean ± standard deviation (s.d.) and range (min–max) are provided.

Variable	Unit	Beluga		Narwhal	
		Mean ± s.d.	Range	Mean ± s.d.	Range
peak	kHz	68.7 ± 10.1	54.4–96.7	43.7 ± 7.8	28.0–58.8
peak2	kHz	67.6 ± 7.9	50.9–89.5	49.3 ± 6.8	34.9–63.3
peak3	kHz	65.5 ± 10.7	43.0–86.4	46.1 ± 8.1	17.3–65.2
trough	kHz	68.7 ± 8.9	51.3–90.4	48.2 ± 6.5	32.1–64.3
trough2	kHz	63.8 ± 9.4	42.4–77.4	44.2 ± 9.5	16.8–65.1
peakToPeak2	kHz	16.6 ± 2.4	13.3–24.0	19.4 ± 2.8	11.0–25.1
peakToPeak3	kHz	16.4 ± 2.4	11.6–22.2	15.3 ± 2.6	5.8–21.2
peak2ToPeak3	kHz	23.5 ± 3.1	17.2–31.4	19.4 ± 4.8	6.2–31.8
Q_10dB	kHz	6.1 ± 4.7	1.7–16.0	3.3 ± 1.4	1.3–10.6
Q_3dB	kHz	33.3 ± 24.7	8.8–93.6	15.0 ± 5.6	6.0–34.8
fmin_10dB	kHz	53.1 ± 8.1	42.4–71.7	33.1 ± 5.5	22.2–51.3
fmin_3dB	kHz	65.0 ± 9.7	50.1–88.1	40.5 ± 6.6	26.3–57.0
fmax_10dB	kHz	87.4 ± 15.2	61.7–119.0	59.3 ± 13.8	33.9–90.0
fmax_3dB	kHz	74.2 ± 12.4	54.3–103.3	47.1 ± 9.3	29.6–66.6
BW_10dB	kHz	34.3 ± 15.9	9.3–65.5	26.2 ± 12.1	10.4–58.8
BW_3dB	kHz	9.2 ± 6.9	0.9–25.3	6.5 ± 3.9	1.9–17.8
centerHz_10dB	kHz	70.2 ± 9.2	53.1–93.6	46.2 ± 8.5	28.1–65.5
centerHz_3dB	kHz	69.6 ± 10.6	52.2–95.7	43.8 ± 7.8	28.1–58.9
duration	µs	659.7 ± 479.9	53.4–1487.0	592.9 ± 488.0	15.0–1667.1
ici	ms	177.9 ± 174.6	2.9–696.5	143.5 ± 70.1	20.3–334.2

### Species acoustic differentiation

**Figure 2:**
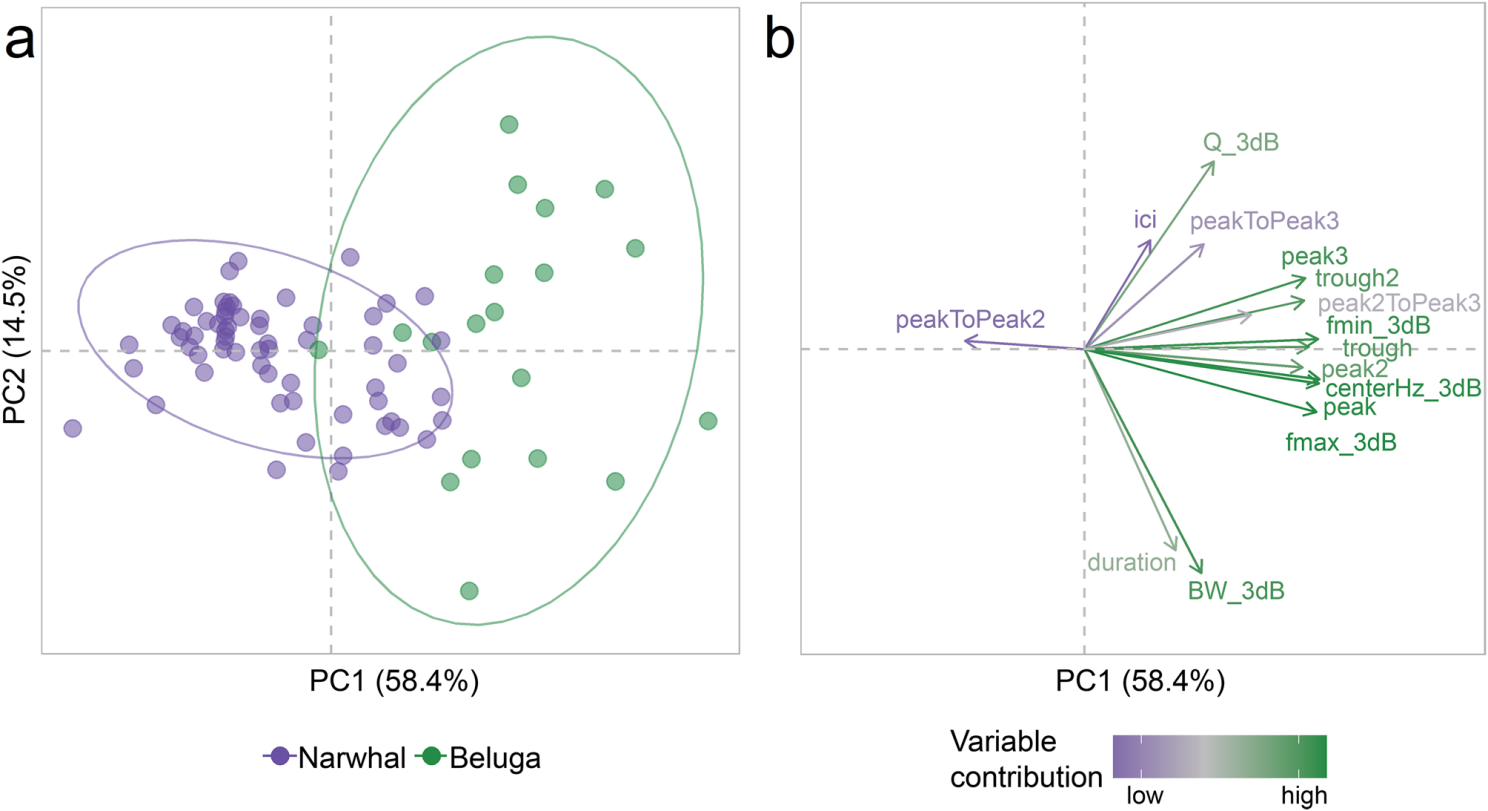
Multivariate PCA ordination plot for beluga or narwhal acoustic events (**a**) and acoustic parameter eigenvectors (**b**). Principle component (PC) 1 explains 58.4% of the variation and PC2 explains 14.5%. The total variable contribution is shown in vector length and color, where purple is a low contribution and green is high. Ellipses in (**a**) show 95% confidence intervals. (**a**) and (**b**) represent the same multidimensional space.

**Figure 3:**
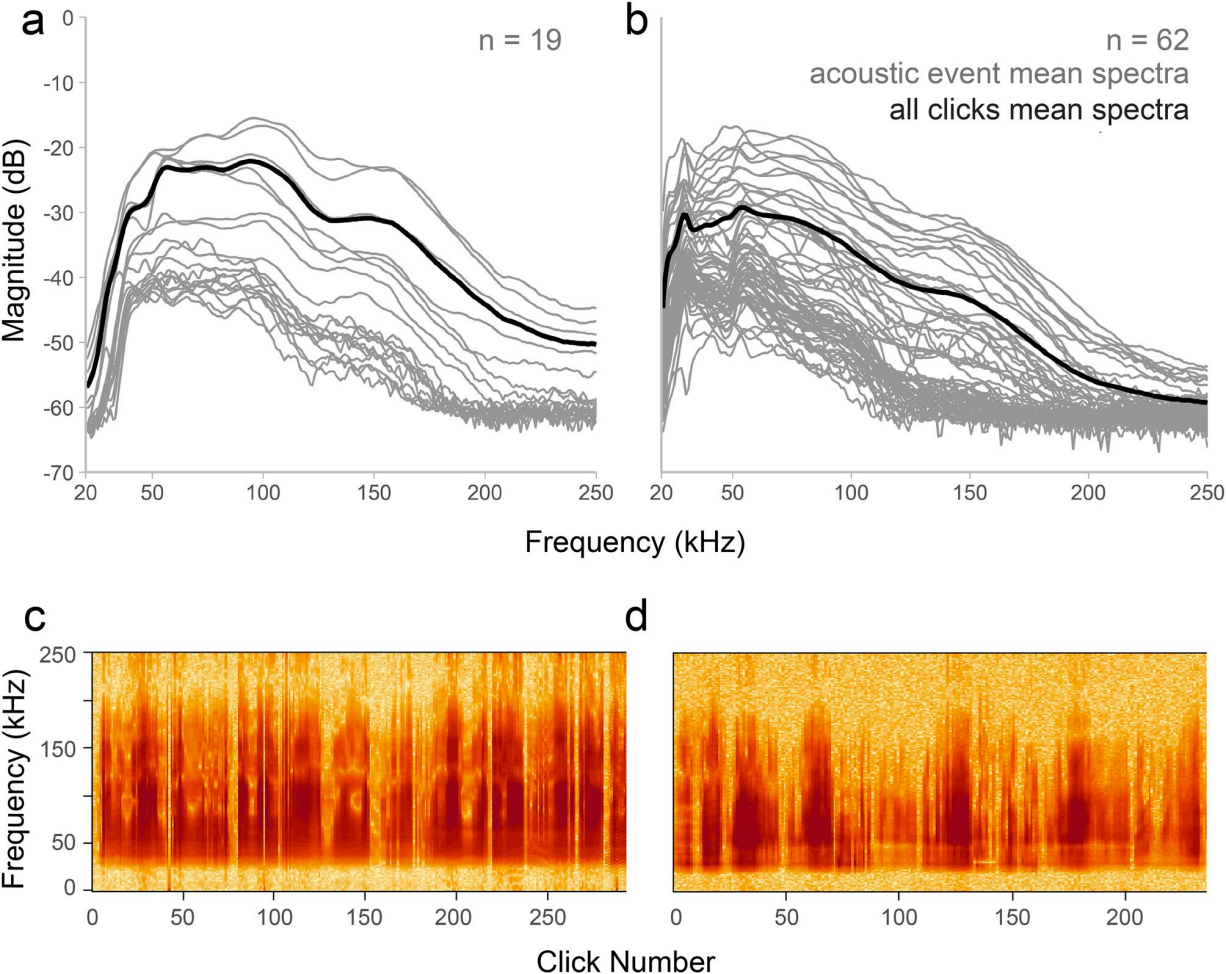
Average spectra and example concatenated spectrograms for beluga (**a**,**c**) and narwhal (**b**,**d**) acoustic events. Gray lines in (**a**) and (**b**) show average spectra for individual acoustic events: 19 beluga events and 62 narwhal events. Black lines in (**a**) and (**b**) represent the mean spectra for all echolocation clicks assigned to events for each species: 2537 beluga clicks and 8782 narwhal clicks. Example concatenated spectrograms in (**c**) and (**d**) show one acoustic event for each species (1024 point FFT; Hanning window).

Results from the perMANOVA reveal significant differences between beluga and narwhal echolocation characteristics (*F*_1,79_ = 42.35, *p* = 0.001; Supplementary Table S2). Beluga acoustic events also demonstrated a higher dispersion in multivariate space than narwhal acoustic events (PERMDISP, *F*_1,79_ = 8.37, *p* = 0.004; Supplementary Table S2). The PCA further displayed strong evidence for beluga and narwhal acoustic differentiation; beluga events were more dispersed than narwhal events (Fig. 2a). Within the two-dimensional space of PC1 and PC2, 72.9% of the original trait variation was explained where most was captured by the first dimension (58.4%; Fig. 2a). According to the broken-stick model, PC1 was the only dimension found to explain a significant amount of the variation in the original data. Beluga acoustic events are largely located on the right side of the PCA ordination space, representing higher echolocation click parameter values as compared to narwhal acoustic events that are on the left side (Fig. 2b; Supplementary Fig. S1). These findings are consistent with average spectra, acoustic event spectrograms, and mean parameter values for each species where beluga spectra present more energy at higher frequencies than narwhal spectra (Fig. 3; Table 2).

### Species acoustic classification

**Figure 4:**
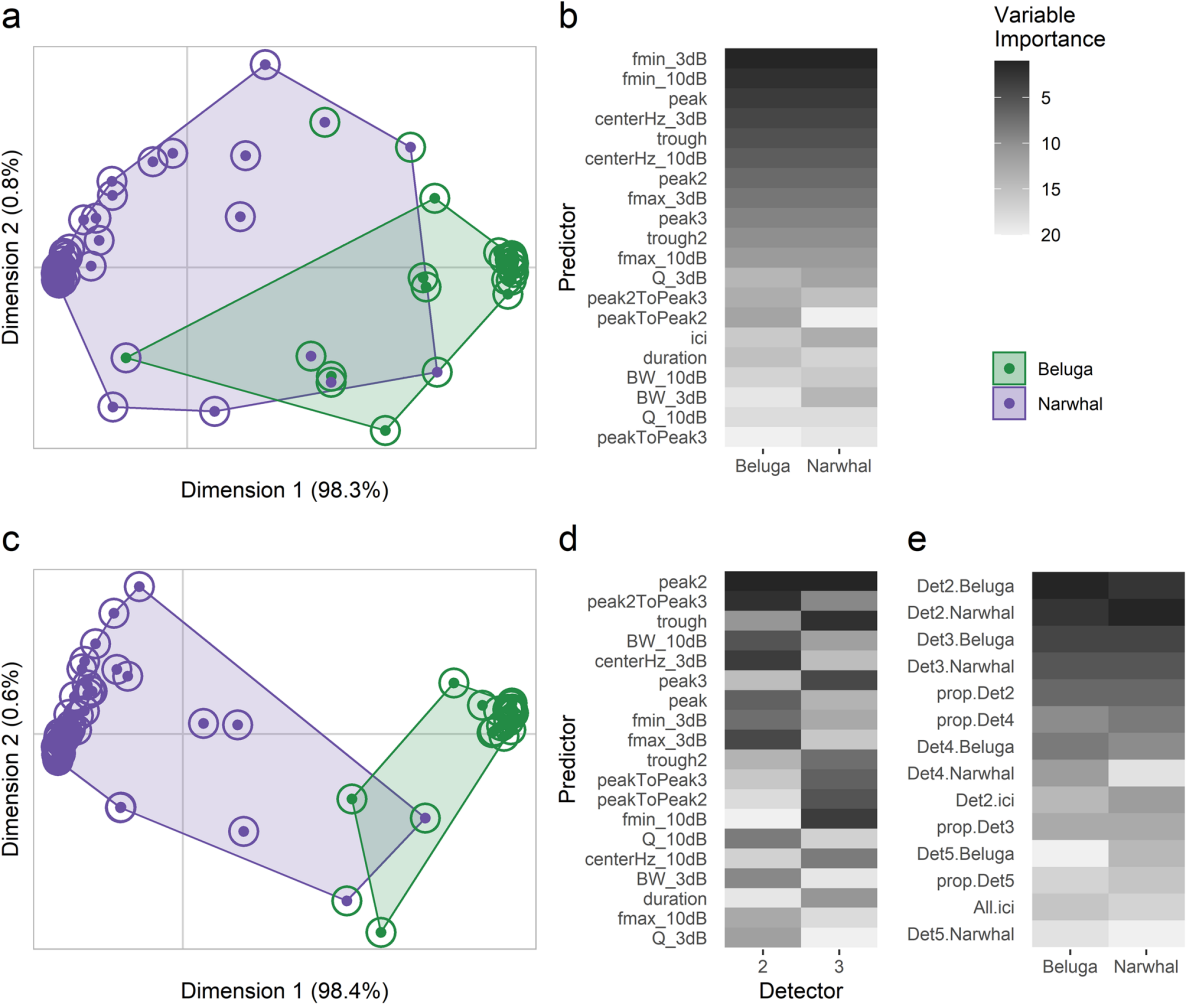
Ordination plots (**a**,**c**) from a multidimensional scale (MDS) conversion of Random Forest (RF) proximity scores to visualize species classification predictions. The top panel (**a**,**b**) corresponds to the single RF classification model and the bottom panel (**c**–**e**) are the results from the BANTER classification model. RF proximity scores are calculated for each pair of objects (i.e., acoustic events) to produce a N × N matrix, where N is the total number of objects. If objects occupy the same terminal node for one tree, their proximity score increases by one. All proximities are normalized by dividing by the total number of trees in the forest. Shaded regions show a-priori species groups with points colored according to their original (inner) and predicted (outer) species. Dimension 1 in (**a**) and (**c**) explains 98.3% and 98.4% of the total variation for the RF and BANTER models, respectively, and dimension 2 explains 0.8% and 0.6% of the variation, respectively. Relative variable importance is shown for both models (**b**,**d**,**e**). The heatmaps show ranked variable importance from the least important (light gray) to most important (dark gray). Descriptions of predictors are provided in Table 1 and Supplementary Table S3.

**Figure 5:**
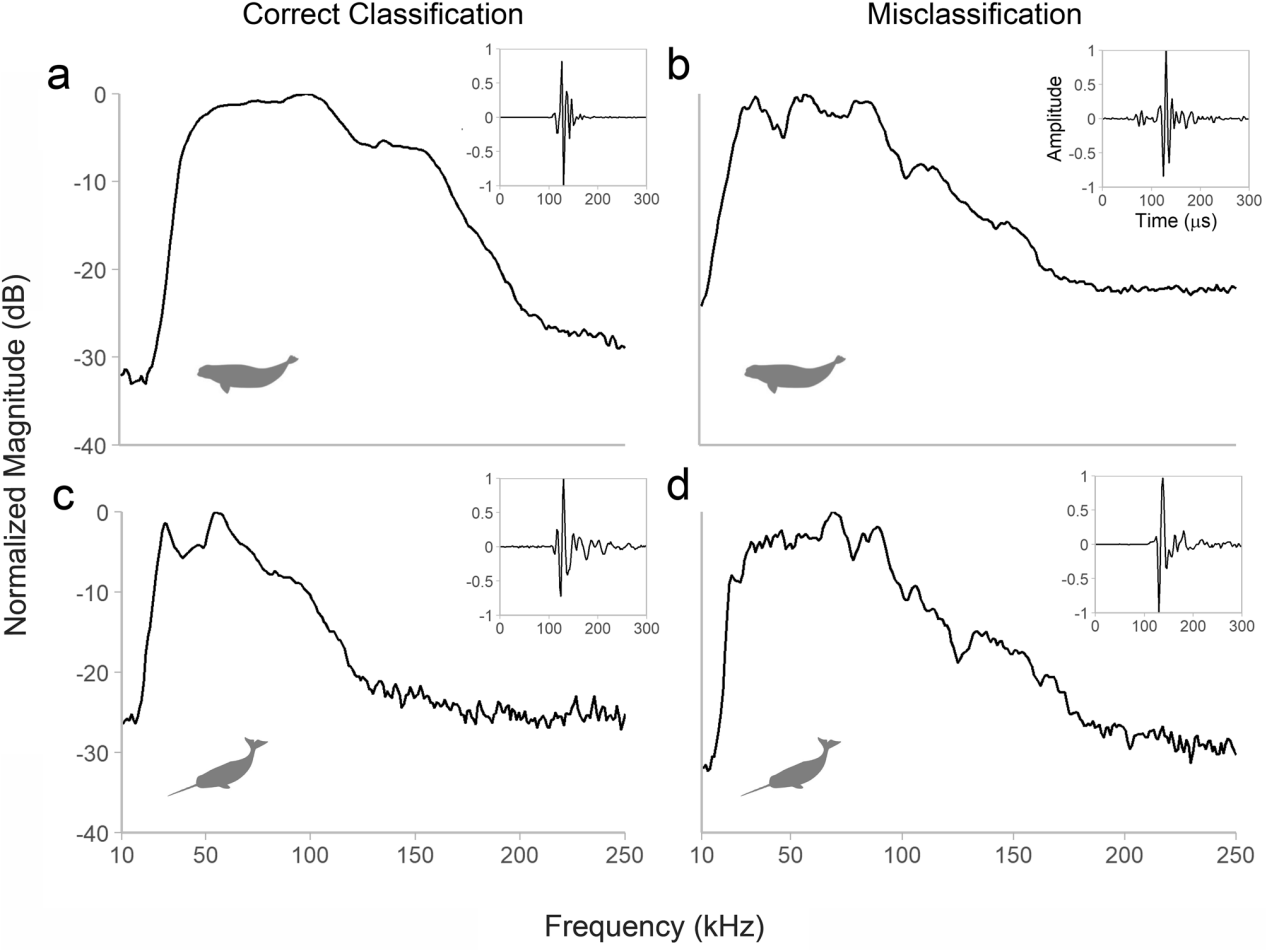
Example average spectra for beluga (**a**,**b**) and narwhal (**c**,**d**) acoustic events that were correctly classified (**a**,**c**) and misclassified (**b**,**d**). The waveforms of the highest amplitude click for each event are provided. The beluga acoustic event in (**b**) was misclassified by only the RF model and the narwhal acoustic event in (**d**) was misclassified by both the RF and BANTER classification models.

Both the RF and BANTER species classification models performed well with high OOB correct classification rates (*p* < 0.001 for all models; Table 3). The overall correct classification rate for the RF model was 92.6% (95% CI: 84.6–97.2%); 94.7% correct classification for beluga acoustic events and 91.9% for narwhal events. One beluga and five narwhal acoustic events were misclassified (Fig. 4a) and the most important predictors for species classification were − 3 dB and − 10 dB frequency minimums and peak frequency (Fig. 4b). Average spectra and waveforms for misclassified events presented signal patterns characteristic of off-axis clicks (i.e., clicks recorded away from the whale’s longitudinal acoustic axis) when compared to those that were correctly classified (Fig. 5).

**Table 3:** Confusion matrices for the (a) standard Random Forest model, (b) BANTER call classifiers (Detectors 2, 3, 4, and 5), and (c) BANTER event classifier. The total number of correct and incorrect classifications are shown between the groups identified a-priori (rows) and predictions by the classifier (columns). Out-of-bag (OOB) percent correct classification rates (95% confidence interval) demonstrate model performance. Binomial test *p*-values using a prior probability of 0.5 are provided where an asterisk (*) represents statistical significance (*p* < 0.05).

	Beluga	Narwhal	Correct classification (95% CI)	*p*-value
**(a) Standard Random Forest model**				
Beluga	18	1	94.7% (74.0–99.9%)	< 0.001*
Narwhal	5	57	91.9% (82.2–97.3%)	< 0.001*
Overall			92.6% (84.6–97.2%)	< 0.001*
**(b) BANTER call classifier**				
Detector 2				
Beluga	351	31	91.9% (88.7–94.4%)	< 0.001*
Narwhal	886	5022	85.0% (84.1–85.9%)	< 0.001*
Overall			85.4% (84.5–86.3%)	< 0.001*
Detector 3				
Beluga	1007	122	89.2% (87.2–90.9%)	< 0.001*
Narwhal	540	1632	75.1% (73.3–76.9%)	< 0.001*
Overall			80.0% (78.5–81.3%)	< 0.001*
Detector 4				
Beluga	710	136	83.9% (81.3–86.3%)	< 0.001*
Narwhal	121	449	78.8% (75.2–82.1%)	< 0.001*
Overall			81.9% (79.7–83.8%)	< 0.001*
Detector 5				
Beluga	131	39	77.1% (70.0–83.1%)	< 0.001*
Narwhal	22	72	76.6% (66.7–84.7%)	< 0.001*
Overall			76.9% (71.3–81.8%)	< 0.001*
**(c) BANTER event classifier**				
Beluga	19	0	100% (82.4–100%)	< 0.001*
Narwhal	2	60	96.8% (88.8–99.6%)	< 0.001*
Overall			97.5% (91.4–99.7%)	< 0.001*

BANTER’s two-stage approach achieved a higher accuracy than the standard RF model. The BANTER first stage call classifier used here consisted of four separate models for click detectors 2, 3, 4, and 5 (Table 3), each producing mean species assignment probabilities and click detector proportions that were subsequently used as variables for the second stage event classifier (Supplementary Table S3). BANTER’s event classifier achieved a correct classification score of 97.5% (95% CI: 91.4–99.7%); 100% correct classification for beluga acoustic events and 96.8% for narwhal events (Table 3). Among the event-level variables, detectors 2 (20–50 kHz) and 3 (50–70 kHz) were the most important classifiers (Fig. 4e). By examining the relative importance of the predictors (i.e., acoustic parameters; Table 1) for detector 2 and 3 call classifiers, the second peak frequency, the frequency difference between the second and third peaks, and the frequency trough were the most important for classification of individual clicks (Fig. 4d). However, since the event classifier uses mean assignment probabilities from each detector classifier as predictors, the most important variables for the detector 2 and 3 classifiers are not necessarily important variables for the event classifier. Examination of the vote distributions for the detector 2 and 3 call classifiers revealed that the detector 2 call classifier produced confident species classifications (i.e., the majority of samples were classified at high probability) compared to the detector 3 classifier (Supplementary Fig. S2). Overall, the BANTER model demonstrated strong predictive power with only two narwhal event misclassifications (Table 3; Fig. 4c). For correctly classified events, the model had high confidence for its species assignment evidenced by its vote distribution (Supplementary Fig. S3).

The correlation test to examine similarity between acoustic encounters revealed that events among different encounters were similar. A positive correlation between training confusion matrices (from models built using one encounter per species) and validation confusion matrices (from training models that predicted events from encounters not used in training model) indicated similarity between encounters. With the exception of one pair, all encounter pairs had a positive correlation and 6 of the 10 pairs had a correlation greater than 0.5 (Supplementary Table S4).

For both the single RF and BANTER models, the majority of the predictions for beluga and narwhal acoustic events were tightly clustered (Fig. 4a,c). These tightly clustered, correctly classified events were marked by clear differences between beluga and narwhal spectral characteristics. Beluga average spectra tended to be smoother, have a larger bandwidth, and higher -3 and -10 dB frequency minimums and maximums (Figs. 3a and 5a). Conversely, narwhal average spectra presented peaks and notches, a lower bandwidth, and lower frequency minimums and maximums (Figs. 3b and 5c). Average spectra from events that were misclassified and showed properties of both beluga and narwhal spectral characteristics generally had lower signal magnitudes (dB), smaller click sample sizes, and spectral variations that suggest a higher proportion of off-axis clicks (i.e., clicks not centered on the recording equipment; Figs. 3 and 5).

## Discussion

Passive acoustics has been a primary method to monitor beluga and narwhal populations year-round in remote regions of the Arctic, providing insight into their seasonal distribution and migratory routes^16,17,33,38,44^. Yet, prior to this study, the acoustic profiles of these two species were examined separately, despite their shared habitat ranges and acoustic features. Our results provide the first acoustic comparison between belugas and narwhals from the same region and reveal that they can be acoustically differentiated and classified using echolocation clicks alone. Belugas and narwhals have considerable acoustic overlap and variability in their social calls which makes species classification using acoustic parameters challenging^17,23,27,32^. Our analysis of beluga and narwhal recordings from the same region, during the same season, and acquired using the same equipment delivers a rare in situ comparison that fills a critical knowledge gap in the acoustic ecology for each species. We also provide a robust BANTER classification model that sets a precedent and foundation for echolocation parameters to be used in future PAM classification efforts, whether for belugas and narwhals or other odontocete species.

Our multivariate analyses showed significant differences between beluga and narwhal echolocation characteristics (perMANOVA: *p* = 0.001) where beluga acoustic parameters were more dispersed and distinguished by higher frequency content (Figs. 2 and 3). Spectral peaks, troughs, and center frequencies for beluga clicks tended to be > 60 kHz and narwhal clicks < 60 kHz with overlap between 40–60 kHz (Table 2). The greater variation observed among beluga acoustic events may be due to the sample size being smaller (*n* = 19) than the narwhal dataset (*n* = 62) or may reflect the true variation in beluga echolocation. Additionally, the lower frequency content observed in narwhal echolocation may indicate that narwhals have a longer echolocation detection range for prey and PAM receivers than belugas. Previous work to quantify beluga echolocation parameters reveals high variability and evidence of biosonar adaptability^16,36,37,40,79^. For example, estimates of beluga echolocation peak frequencies range widely between 40–120 kHz across captive and wild environments^34,36–38,40,79^ and have been shown to change depending on the ambient sound levels of the environment^36^. Narwhal echolocation has been largely characterized by lower frequency clicks with maxima between 30–70 kHz^30,32,43,44^, but high-frequency clicks have been reported where the entire bandwidth can extend up to 200 kHz^25^. Frouin-Mouy et al*.*^17^ used a difference in spectral power around the 20 kHz frequency band to distinguish between species for mid-frequency clicks with peaks within 30–60 kHz^17^. However, their sample sizes were small with 20 beluga and 17 narwhal click trains (162 and 186 individual clicks, respectively), and their recordings came from five different locations and three different receivers which can introduce classification errors^35^. Our findings are consistent with their preliminary comparison in which narwhal spectra contain more energy between 20–50 kHz than beluga spectra (Fig. 3). Yet, it remains unknown to what degree the variation observed in beluga and narwhal echolocation is due to context-specific active biosonar adjustments made by the whales, the orientation of whales relative to the receiver, differences in sound propagation in the water, population- or individual-specific characteristics, and/or differences in sampling design, recording equipment, or data processing.

Between the two classification models presented in this study, the BANTER model proved to be the strongest classifier with a correct classification rate of 97.5%. The BANTER event classifier accurately predicted all beluga events (100%) and misclassified only two narwhal events (3.2%; Table 3). As a balanced design, the BANTER classifier does not bias towards either species, but it reveals that beluga events are more diagnosable evidenced by the correct prediction of all beluga events by the event classifier. This may occur because a higher proportion of the beluga events—the smaller sample size group—was randomly selected for the training dataset compared to the narwhal group across all *n* trees. Results from BANTER’s two-stage approach identifies which frequency ranges contain the most useful information for species classification. The call classifiers for detectors 2 (20–50 kHz) and 3 (50–70 kHz) were found to be the most important for BANTER’s event classifier, suggesting clicks with peak frequencies between 20–70 kHz largely contained the information necessary for correct species assignment to acoustic events (Fig. 5a,c). Higher frequencies attenuate faster than lower frequencies^1^; therefore, it is possible that the lower frequency classifiers (detectors 2 and 3) were the most important because lower frequency signals were detected more than higher frequency signals. These results also indicate that the BANTER model will be less affected by variations in detection distances between species that would more largely influence the higher frequency detector 4 and 5 classifiers.

While BANTER’s call classifiers used acoustic parameters for predictors, the event classifier used mean assignment probabilities and detector proportions for predictors. The most important predictors for the event classifier were the mean assignment probabilities for detector 2 (Fig. 4e). Given the reasonably high overall classification rate (85.4%) and vote distribution for the detector 2 call classifier, it is likely that the most important variables for this classifier were also important for the event classifier. For the detector 2 call classifier, the second peak frequency, frequency difference between the second and third peaks, and − 3 dB center frequency were the most important variables for the classification of clicks (Fig. 4d). Soldevilla et al*.*^80^ demonstrated that Risso’s (*Grampus griseus*) and Pacific white-sided dolphins (*Lagenorhynchus obliquidens*) could be classified using spectral peak and notch patterns from echolocation clicks. Here, we similarly demonstrate the efficacy of using unique spectral properties to classify belugas and narwhals.

Our results from the single RF model demonstrate how a simple RF classifier using only echolocation parameters can still achieve a high correct species classification rate (92.6%). The RF model accurately predicted beluga events more than narwhal events, with one beluga misclassification (5.3%) and five narwhal misclassifications (8.1%; Table 3). Much like the BANTER model, the lower misclassification rate of beluga events may be in part due to the unbalanced dataset; a higher proportion of the beluga events were randomly selected in the RF model because it was the smaller sample size group. However, it is also possible that beluga events contain more diagnosable features and thus yield higher classification scores. The most important acoustic parameters for species classification in the standard RF model differed from those in BANTER’s detectors 2 and 3 call classifiers. Among all 20 acoustic parameters used in the RF model, frequency minimum (− 3 dB and − 10 dB) and peak frequency were the most important variables for species assignment (Fig. 4b).

Using echolocation predictors for species classification is an effective approach, particularly given its primary sensory role for odontocetes. There is evidence for individual and group-specific call types among belugas^81–84^ and narwhals^85^, which contributes to the variation in their vocal repertoire but also makes acoustic classification challenging. However, it is likely that echolocation clicks are characterized by more stable features due to their sensory function. There are occasions where whales are only echolocating and not producing other call types (e.g., whistles, burst pulses)^17^, and as such, a classifier that uses echolocation may offer more reliable year-round detections. While it is likely that the addition of other call types would improve classification^28,35^, the BANTER classifier presented here demonstrates belugas and narwhals can be classified by clicks alone. Echolocation is increasingly being incorporated into PAM classification programs^28,74,80,86^ and is specifically gaining traction for monitoring belugas^16,19,33^. Noise from ice floes, vessels, or industrial activities below 40 kHz can mask lower frequency social calls (e.g., Lammers et al*.*^34^ and Halliday et al*.*^87^). The broadband, high frequency nature of echolocation clicks is largely protected from masking by lower frequency anthropogenic sounds. As vessel traffic is expected to increase in the Arctic, echolocation may be a stronger metric for PAM classification.

Yet, the directionality and high-frequency nature of echolocation clicks pose their own limitations to species detectability and classification. Acoustic receivers will record echolocation sounds when the whale is facing the receiver but are unlikely to detect whales that are pointing their sonar beam away from the recorder. Narwhals are known to forage for Greenland halibut (*Reinhardtius hippoglossoides*) and *Gonatus* squid species at depths often > 1000 m in the winter^88,89^, so they may be detected by seafloor recorders during these months. While belugas can dive to depths > 500 m and reach the seafloor, they do not prey upon benthic species like narwhals; instead, they target fish species like the Arctic cod (*Arctogadus glacialis*) and polar cod (*Boreogadus saida*) that occupy shallower depths^90,91^. Therefore, due to the differences in prey selection by belugas and narwhals, they may not be detected by seafloor recorders with the same confidence depending on the recording depth of the instrument. Additionally, the rapid attenuation of ultrasonic echolocation signals^1^ results in a smaller detection range for echolocation clicks (< 1 km^39^) compared to lower-frequency social calls. Multiple receivers and the inclusion of other call types (e.g., whistles) in the classifier may be required to detect whales at a sufficiently large spatial resolution. In addition to deploying more receivers, the instruments required to record echolocation clicks must record higher frequencies with higher sampling rates, which typically depend on more battery power and memory storage. Ongoing development of acoustic receivers to be both economical and reliable will support monitoring populations using echolocation clicks at large scales.

This study provides a unique case in which the methods employed were identical for each species studied, so confounding variables that can substantially affect parameterization, like differences in the frequency responses of recording systems used^35^, are absent. However, the data used to build the classifiers originated from a limited number of independent acoustic encounters: two beluga and five narwhal. When testing for the similarity between independent acoustic encounters using a BANTER framework, our results indicate that there is likely not a strong effect of encounter on species classification. Additional data from multiple, independent groups of whales are needed to more accurately reflect the true variation in beluga and narwhal echolocation. While we show it is possible to differentiate and classify belugas and narwhals using echolocation clicks, incorporating data from additional independent encounters and other call types will strengthen the classifier against any individual- or pod-specific acoustic characteristics that may be present.

Future work to test the BANTER classification model presented here will elucidate its efficacy for PAM applications. Acoustic recordings used in this study were collected with a hydrophone deployed off the ice-edge positioned at a depth of 12 m. Assuming the new data follow the methodology employed here—including the recording equipment and sampling rate (500 kHz sampling rate; 16-bit resolution)—our model has the potential to be used on novel data. However, it remains unclear how effective the model presented here will be when data collected at depth, such as seafloor recorders where detectability of echolocation may decrease and recordings are expected to have a higher proportion of off-axis clicks, are applied. Additional examination is needed to assess the degree to which differences in recording equipment (e.g., Scripps Institution of Oceanography High-frequency Acoustic Recording Package [HARPs] or Ocean Instruments’ SoundTrap recorders) or receiver depth would affect the outcome of the presented classifier. Likewise, further consideration of which acoustic parameters are more stable across various equipment may mitigate effects of differing platforms^28,35^.

Insufficient data on beluga and narwhal populations has made it difficult to determine major threats to these species and thus hindered specific conservation efforts^92^. Narwhals have been identified as one of the most sensitive Arctic marine mammals to climate change due to their specialized habitat niche and restricted distribution range^93^. Both belugas and narwhals have been shown to have high site fidelity to their summering and wintering grounds, but little is known about what factors (e.g., prey availability or ice conditions) cause these behavior^88,94,95^. Furthermore, global climate warming is causing a lengthened open-water season in the Arctic that has galvanized opportunities for increased hydrocarbon exploration and commercial and recreational shipping development^3,48,92,96^. Some of the largest unexploited hydrocarbon reserves are found in the Arctic, particularly Baffin Bay^97,98^, and most global trade depends on maritime transport. Over half of the distribution ranges of the three endemic Arctic whale species overlaps with known or anticipated offshore hydrocarbon deposits^3^. Stakeholders of international trade and hydrocarbon exploration need to be informed of what regions are critical to belugas and narwhals for vital processes (e.g., calving, feeding, migrating) in order to mitigate industrial activities^17^. Strict regulations on vessel speed, appropriate shipping lanes, and types of permissible activities will be needed to avoid harmful consequences^92^.

As advances in PAM increase efficiency and robustness of data collection and analyses in the Arctic, present and future distributions of belugas and narwhals can be recorded with greater confidence and resolution, thereby improving efforts to monitor their changes in response to climate change and increased underwater noise. The multivariate nature of acoustic parameters lends itself to the interactive effects between variables, making methods like RF classifiers that can handle correlated variables robust for PAM species detection. BANTER increases classification accuracy beyond a standard RF model by using information from misclassifications at the call classification stage to inform the overall event classifier. Results from this study fill a critical data gap in the acoustic ecology of belugas and narwhals and provide a promising approach for future PAM programs. As recording systems advance and training datasets expand, classification programs such as the one presented here will develop greater predictive power and allow acousticians to reliably monitor Arctic odontocete populations long-term. Despite the variation in beluga and narwhal acoustic repertoires, our study suggests that the use of echolocation parameters for species classification may provide the most reliable method to differentiate these iconic species.

## Supplementary Information

Supplementary Information.

## Data Availability

All code used for model design and implementation are freely available via Github: https://github.com/mjzahn/beluga_narwhal_classifier.
