# Supplementary material for: Acoustic differentiation and classification of wild belugas and narwhals using echolocation clicks
Source: Sci Rep. 2021 Nov 12;11:22141. doi: 10.1038/s41598-021-01441-w (PMC8589986; doi:10.1038/s41598-021-01441-w)
Supplement: Supplementary file 1 — Supplementary Information. [file 41598_2021_1441_MOESM1_ESM.pdf]

Supplementary Information

Acoustic differentiation and classification of wild belugas and narwhals using echolocation clicks

Marie J. Zahn[1*], Shannon Rankin[2], Jennifer L. K. McCullough[3], Jens C. Koblitz[4,5,6], Frederick Archer[2], Marianne H. Rasmussen[7], Kristin L. Laidre[1,8]

[1]School of Aquatic and Fishery Sciences, University of Washington, 1122 NE Boat Street, Seattle, Washington 98105, USA

[2]Southwest Fisheries Science Center, NOAA, 8901 La Jolla Shores Drive, La Jolla, California 92037, USA

[3]Pacific Islands Fisheries Science Center, NOAA, 1845 Wasp Boulevard, Building 176, Honolulu, HI, 96818, USA

[4]Max Planck Institute of Animal Behavior, Advanced Research Technology Unit, Konstanz, Germany

[5]Centre for the Advanced Study of Collective Behaviour, University of Konstanz, Konstanz, Germany

[6]Department of Biology, University of Konstanz, Konstanz, Germany

[7]The University of Iceland's research center in Húsavík, Húsavík, Iceland

[8]Polar Science Center, Applied Physics Laboratory, University of Washington, 1013 NE 40th Street, Seattle, Washington, 98105, USA

[*]Corresponding author: mzahn@uw.edu

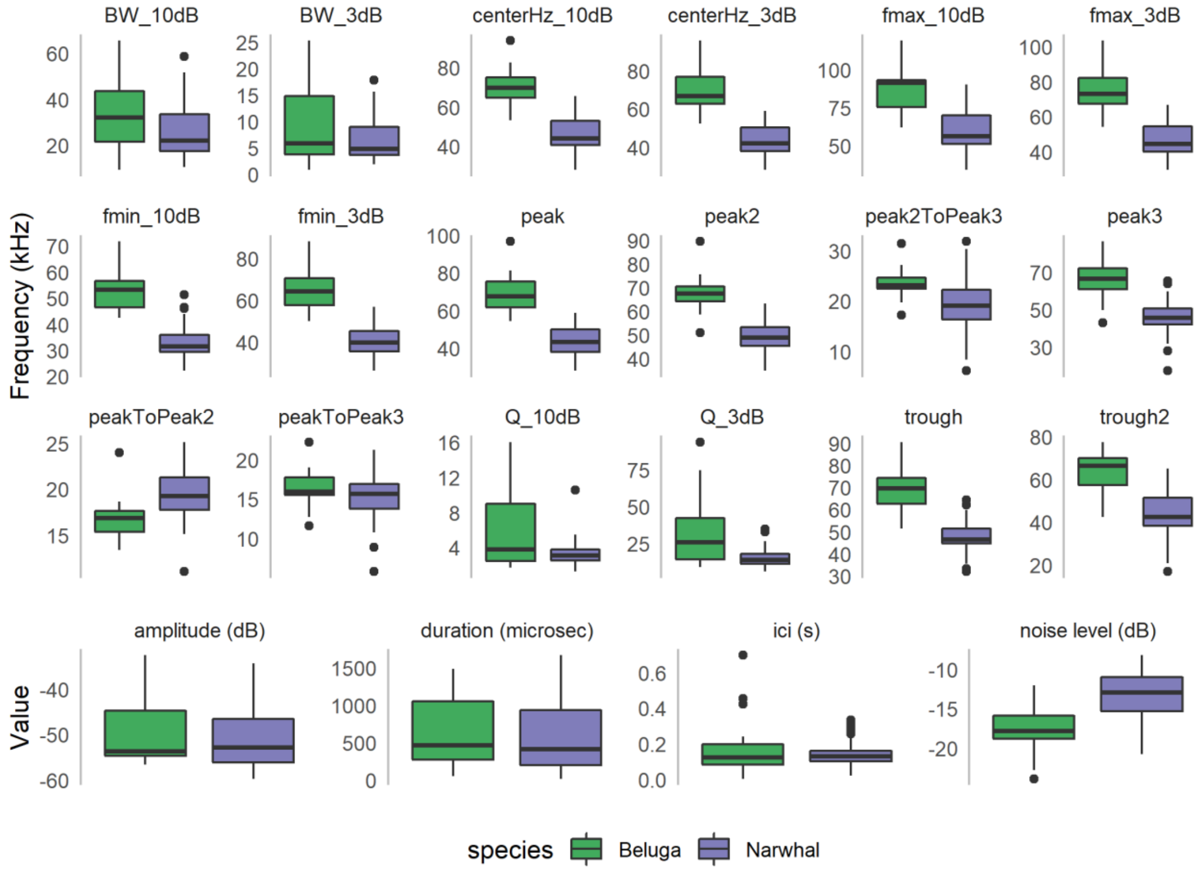

Figure S1. Beluga (green) and narwhal (purple) echolocation parameter variation for each of the variables calculated in the *PAMpal* R package. Click amplitude and noise level were not used in analyses.

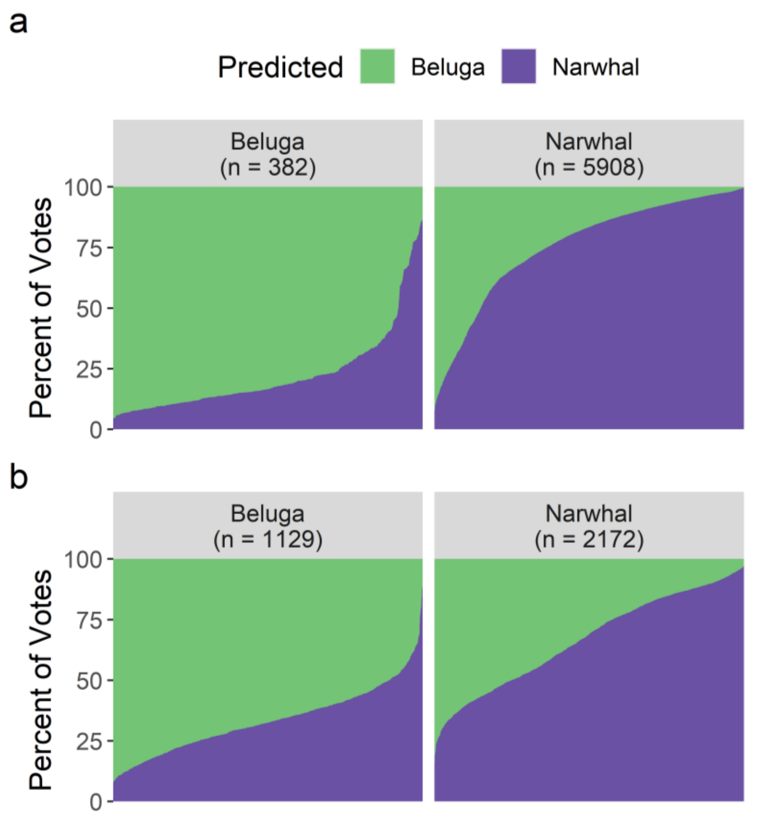

Figure S2. Distribution of model votes (beluga or narwhal classification) for each click that was assigned *a priori* to a species class for the detector 2 (**a**) and detector 3 (**b**) BANTER call classifiers. The distribution demonstrates the overall confidence of the call classifier results.

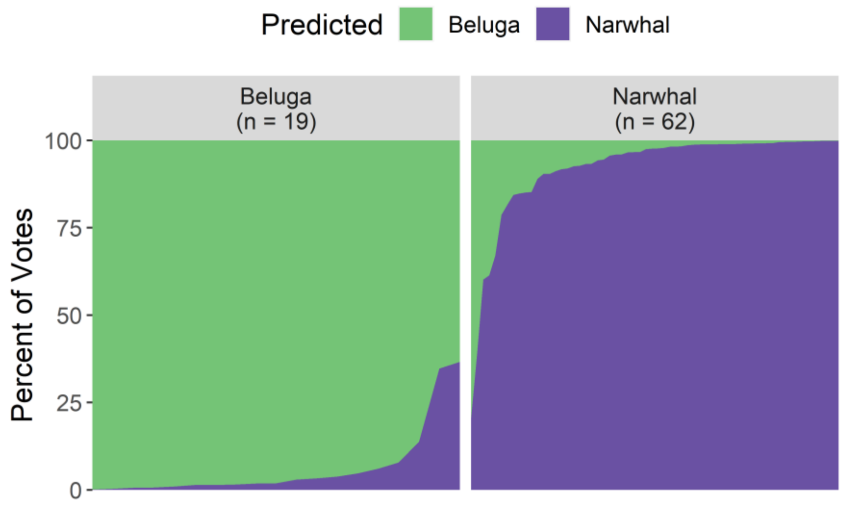

Figure S3. Distribution of model votes (beluga or narwhal classification) for each event that was assigned *a priori* to a species class. The distribution demonstrates the overall confidence of the BANTER event classifier results.

Table S1. Summary of the number of echolocation clicks assigned to each acoustic event in PAMGuard for each species (N = narwhal; B = beluga).

Event	Species	# Clicks	Event	Species	# Clicks
1	N	17	42	N	462
2	N	65	43	N	49
3	N	280	44	N	252
4	N	27	45	N	279
5	N	20	46	N	26
6	N	17	47	N	64
7	N	39	48	N	204
8	N	444	49	N	364
9	N	131	50	N	208
10	N	6	51	N	472
11	N	203	52	N	41
12	N	111	53	N	126
13	N	158	54	N	52
14	N	284	55	N	274
15	N	401	56	N	235
16	N	119	57	N	239
17	N	48	58	N	11
18	N	93	59	N	24
19	N	130	60	N	17
20	N	238	61	N	18
21	N	71	62	N	13
22	N	155	63	B	120
23	N	52	64	B	57
24	N	31	65	B	10
25	N	117	66	B	20
26	N	14	67	B	292
27	N	337	68	B	928
28	N	608	69	B	37
29	N	6	70	B	20
30	N	14	71	B	183
31	N	305	72	B	498
32	N	137	73	B	89
33	N	102	74	B	19
34	N	266	75	B	16
35	N	28	76	B	24
36	N	150	77	B	18
37	N	8	78	B	12
38	N	55	79	B	171
39	N	22	80	B	13
40	N	57	81	B	11
41	N	134			

Table S2. PerMANOVA and PERMDISP test results on z-score transformed data to assess the differences between beluga and narwhal echolocation. The asterisk (*) indicates a statistically significant result ($P < 0.05$).

	df	SSE	MSE	*F*	R^2	*P*-value
PerMANOVA						
Species	1	418.78	418.78	42.35	0.35	0.001*
Residuals	79	781.22	9.89		0.65	
Total	80	1200.00			1.00	
PERMDISP						
Groups	1	12.95	12.95	8.37		0.004*
Residuals	79	122.17	1.55			
Total	80	135.12				

Table S3. Descriptions of predictors used for BANTER's event classifier. Values for these predictors are determined from BANTER's call classifier. The 'X' in variable codes is a placeholder for either 'beluga' or 'narwhal.'

Variable Code	Description
Det2.X	Mean probability that Detector 2 clicks (20–50 kHz) for a given event will be assigned to species X
Det3.X	Mean probability that Detector 3 clicks (50–70 kHz) for a given event will be assigned to species X
Det4.X	Mean probability that Detector 4 clicks (70–100 kHz) for a given event will be assigned to species X
Det5.X	Mean probability that Detector 5 clicks (100–150 kHz) for a given event will be assigned to species X
prop.Det2	Proportion of Detector 2 clicks (20–50 kHz) for a given event
prop.Det3	Proportion of Detector 3 clicks (50–70 kHz) for a given event
prop.Det4	Proportion of Detector 4 clicks (70–100 kHz) for a given event
prop.Det5	Proportion of Detector 5 clicks (100–150 kHz) for a given event
Det2.ici	Time interval (in sec) between consecutive clicks for only Detector 2 clicks; mode approximated for each event
All.ici	Time interval (in sec) between consecutive clicks across all detectors; mode approximated for each event

Table S4. Results for the correlation test to examine the similarity between independent acoustic encounters. Each row corresponds to a separate BANTER classification model that used one encounter for each species as training data (10 unique combinations). The number of events comprising each encounter and out-of-bag (OOB) percent correct classification rates (95% confidence interval) for model performance are reported. The correlation between the validation confusion matrices (made predictions on encounters that were not used in the training model) and the training model confusion matrices indicates degree of similarity between encounters.

Encounter ID		# Events		Training model correct classification (95% CI)	Confusion matrix correlation
Beluga	Narwhal	Beluga	Narwhal		
2	4	16	2	94.4% (72.7 – 99.9%)	-0.54
2	3	16	8	95.8% (78.9 – 99.9%)	0.08
2	6	16	8	91.7% (73.0 – 99.0%)	0.12
7	4	3	2	100% (47.8 – 100%)	0.37
2	5	16	14	100% (88.4 – 100%)	0.53
7	5	3	14	100% (80.5 – 100%)	0.63
2	1	16	30	97.8% (88.5 – 99.9%)	0.82
7	6	3	8	90.9% (58.7 – 99.8%)	0.83
7	1	3	30	100% (89.4 – 100%)	0.96
7	3	3	8	100% (71.5 – 100%)	0.98
